# Improved survival of autologous stem cell transplantation in primary refractory and relapsed Hodgkin lymphoma in the brentuximab vedotin era — real-world data from Hungary

**DOI:** 10.1007/s00277-023-05354-8

**Published:** 2023-07-10

**Authors:** Kata Husi, Roxána Szabó, László Imre Pinczés, Dóra Földeák, Réka Dudley, Árpád Szomor, Beáta Koller, László Gopcsa, Árpád Illés, Zsófia Miltényi

**Affiliations:** 1 Division of Hematology, Department of Internal Medicine, Faculty of Medicine, University of Debrecen, Nagyerdei Krt. 98, 4032 Debrecen, Hungary; 2 Doctoral School of Clinical Medicine, University of Debrecen, Debrecen, Hungary; 3 Division of Hematology, 2nd Department of Internal Medicine, Faculty of Medicine, University of Szeged, Szeged, Hungary; 4 Division of Hematology, 1st Department of Internal Medicine, Faculty of Medicine, University of Pécs, Pécs, Hungary; 5 Department of Hematology and Stem Cell Transplantation, Central Hospital of Southern Pest National Institute of Hematology and Infectious Diseases, Budapest, Hungary

**Keywords:** Hodgkin lymphoma, Autologous stem cell transplantation, Brentuximab vedotin, PET/CT, Survival

## Abstract

Autologous stem cell transplantation (ASCT) is the standard treatment of primary refractory or relapsed Hodgkin-lymphoma, which can provide a cure rate of about 50%. The aim of our study was to analyze the data of 126 HL patients undergoing AHSCT in Hungary between 01/01/2016 and 31/12/2020. We assessed the progression-free and overall survival, the prognostic role of PET/CT performed before transplantation and effect of brentuximab vedotin (BV) treatment on survival outcomes. The median follow-up time from AHSCT was 39 (1–76) months. The 5-year OS comparing PET- and PET + patients was 90% v. 74% (*p* = 0.039), and 5-year PFS was 74% v. 40% (*p* = 0.001). There was no difference in either OS or PFS compared to those who did not receive BV before AHSCT. We compared BV treatments based on their indication (BV only after AHSCT as maintenance therapy, BV before and after AHSCT as maintenance treatment, BV only before AHSCT, no BV treatment). There was statistically significant difference in the 5-year PFS based on the inication of BV therapy. Recovery rates of our R/R HL patient population, who underwent AHSCT, improved significantly. Our positive results can be attributed to the PET/CT directed, response-adapted treatment approach, and the widespread use of BV.

## Introduction

Today, 80–90% of Hodgkin lymphoma (HL) patients can be cured [1, 2]. However, approximately 20–30% of patients are refractory to first-line therapy or relapse later. Currently, autologous hematopoietic stem cell transplantation (AHSCT) is the standard treatment in primary refractory or relapsed (R/R) cases, which can provide a cure rate of about 50% [3–9]. Adverse predictors of post-AHSCT outcome include primary refractory disease, short (< 12 months) first complete remission, extranodal disease, bulky lesions, poor performance status, and, particularly, the persistence of metabolically active tumor burden on pre-AHSCT fluorine-18-fluorodeoxyglucose positron emission tomography/computed tomography (PET/CT) scan [6, 11, 12]. Over recent years, several strategies have been assessed to improve post-AHSCT outcomes and augment HL cure rates. Unfortunately, many of these approaches showed less than satisfactory results until the introduction of brentuximab vedotin (BV), a conjugate of an anti-CD30 monoclonal antibody, and the microtubule-disrupting agent, monomethyl auristatin E. The AETHERA trial established BV as a consolidation treatment option for patients at high risk of progression or relapse after AHSCT [12]. BV consolidation has been authorized by regulatory approval in Hungary since 2016. BV can also be used in R/R cases after at least two other therapies, when autologous stem cell transplant is not an option (in these cases AHSCT is most often not an option because the disease is not in remission). Although there is no clear recommendation for the subsequent treatments, BV became a part of salvage treatments in everyday practice. Mostly BV combined with bendamustine, DHAP (dexamethasone, cytarabine, and cisplatin), ESHAP (etoposide, methylprednisolone, cytarabine, and cisplatin), ICE (ifosfamide, carboplatin, and etoposide), or IGEV (ifosfamide, gemcitabine, and vinorelbine). Also, based on the results of the ECHELON-1 study, BV is now available in the first-line treatment of advanced-stage disease, combined with AVD (doxorubicin, vinblastine, and dacarbazine) [13]. As a result of this change in practice, the proportion of patients who received BV treatment before AHSCT is rising.

This study aimed to assess the progression-free (PFS) and overall survival (OS) of all HL patients who underwent AHSCT at the four national transplantation centers in Hungary, and also to investigate the prognostic role of PET/CT performed before transplantation, with particular emphasis on pre-AHSCT disease status and use of BV therapy. We compared our results with other studies in adult Hodgkin lymphoma, regarding progression-free and overall survival after transplantation, the depth of remission before transplantation, and the timing of brentuximab vedotin treatment.

## Patients and methods

We retrospectively analyzed the data of HL patients undergoing AHSCT in Hungary between 01/01/2016 and 31/12/2020. Patients were treated in accordance with the evidence- and consensus-based practice guidelines of the Hungarian Society of Haematology and Transfusion [14]. Eligible patients had a histologically confirmed diagnosis of classical cHL and were 18 years or older. Response to therapy was assessed using the 2016 Refinement of the Lugano Classification Lymphoma Response Criteria [15].

All patients received ABVD (doxorubicin, bleomycin, vinblastine, and dacarbazine) polychemotherapy as a first-line treatment per the national guidelines. R/R patients received two cycles of DHAP polychemotherapy as the first salvage treatment, followed by a PET/CT scan. PET/CT-negative patients (Deauville score 1–3) underwent AHSCT at any time after cycle 2, while PET/CT-positive (Deauville score 4–5) patients were administered further salvage therapy. The second salvage protocols were two cycles of BV-based, combined immunochemotherapy, including BV-bendamustine, BV-ICE, and BV-IGEV. A dedicated PET/CT scan was performed after the second cycle of BV-based therapy. AHSCT was performed in case of PET/CT negativity. In PET/CT-positive cases, further salvage treatment or receiving AHSCT (with the determination of further treatment after transplantation) was the transplant center’s sovereign decision. Stem cell mobilization and collection, also the administration of standard supporting treatment, were performed according to institutional guidelines.

Maintenance BV treatment was applied if one or more of the following criteria were met, according to the modified AETHERA criteria: [1] primary refractory HL (defined as progression during or failure to achieve a complete remission after frontline therapy) or relapse within 12 months after first-line therapy, [2] extranodal disease at relapse, [3] B-symptoms at relapse, [4] ≥ 2 prior salvage therapies, [5] partial response (PR) or stable disease (SD) to most recent salvage therapy [12].

The overall survival (OS) was calculated from the day of AHSCT to the last follow-up visit or death. Progression-free survival (PFS) was defined as the time from AHSCT to disease progression or death. Continuous variables are given as their medians and ranges, while categorical variables are with frequencies and percentages. Data normality was evaluated by the Kolmogorov–Smirnov test. Life-table estimates for OS and PFS were calculated by the Kaplan–Meier method, and the log-rank test was used to compare survival curves between groups. The level of statistical significance was considered at *p* < 0.05. Statistical analyses were performed using SPSS 26.0 (IBM Corp., Armonk, NY, USA).

## Results

### Patient characteristics and treatment

A total of 126 HL patients did undergo AHSCT during the examined period and were therefore eligible for inclusion in our study (Table 1). Median age at HL diagnosis was 30 (15–61) years, and 34 (18–63) at the time of AHSCT. Most patients (57%) were males, with nodular sclerosis as the most common histological subtype (68%). A slight majority (53%) of the patients were refractory to primary treatment, and 68% received two or more salvage treatments, all of them receiving BV therapy prior to AHSCT. In 28 (24%) patients, AHSCT was performed with metabolically active tumor burden on pre-AHSCT PET/CT scan. The median follow-up time after AHSCT was 39 (1–76) months. One hundred seven patients were alive at the time of data analysis, while 11 patients died, and 8 were lost to follow-up. The median number of BV consolidation cycles was 8.5 [1–16]. The most common side effect was peripheral sensory neuropathy, but investigation of side effects was not an aim of this study.

**Table 1:** Patient characteristics and treatment

	Patients	%
Female	54	43
Male	72	57
*Histological subtypes*		
MC	18	14
NS	86	68
LR	4	3
LD	5	4
NLPHL	5	4
ND	7	5
NS-LD	1	
*Disease status after first-line treatment*		
Refractory	67	53
Relapse < 12 months	33	26
Relapse ≥ 12 months	22	17
Unknown	4	4
*Number of salvage treatment*		
1	40	32
2 or more	86	68
*Pre AHSCT PET/CT status*		
PET − (Deauville 1–3)	88	76
PET + (Deauville 4–5)	28	24
*Post AHSCT PET/CT status (100 days)*		
PET − (Deauville 1–3)	75	77
PET + (Deauville 4–5)	22	23
* Relapse after AHSCT*	35	27

### Outcome

**Fig. 1:**
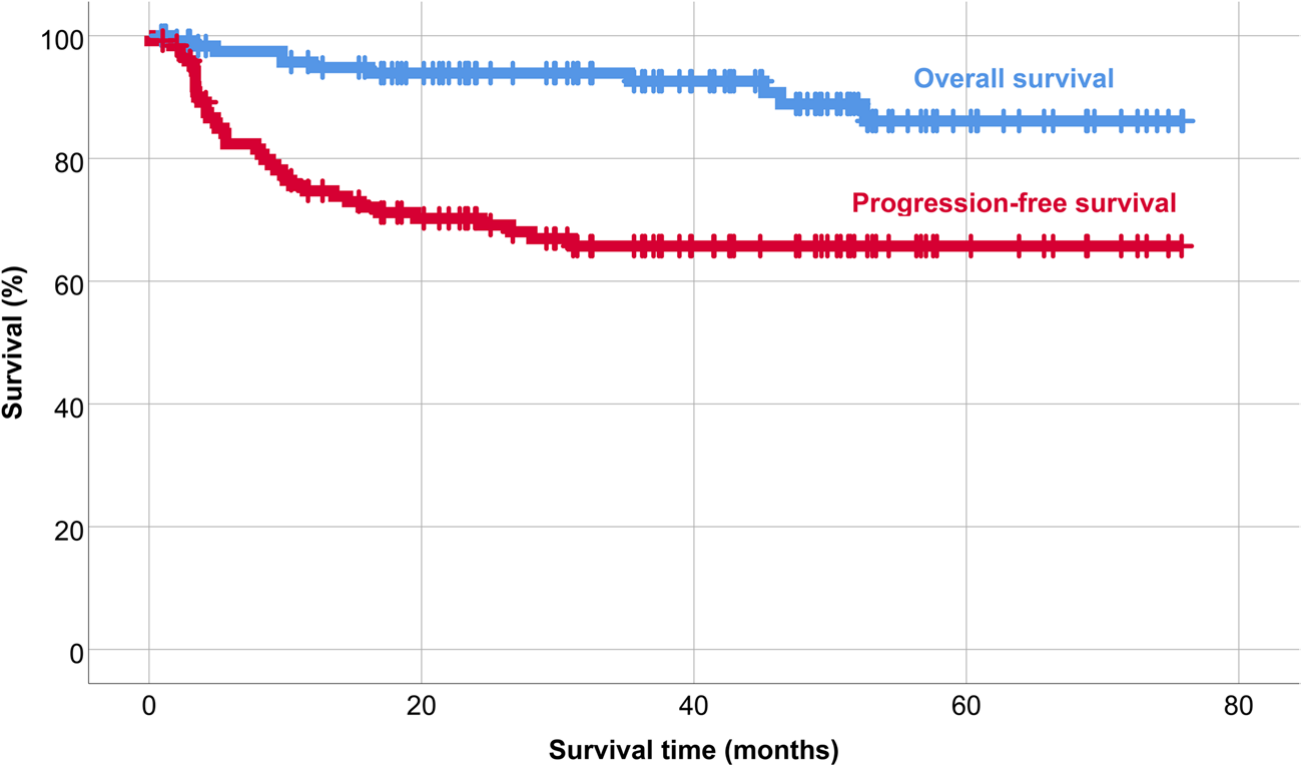
Overall and progression-free survival

**Fig. 2:**
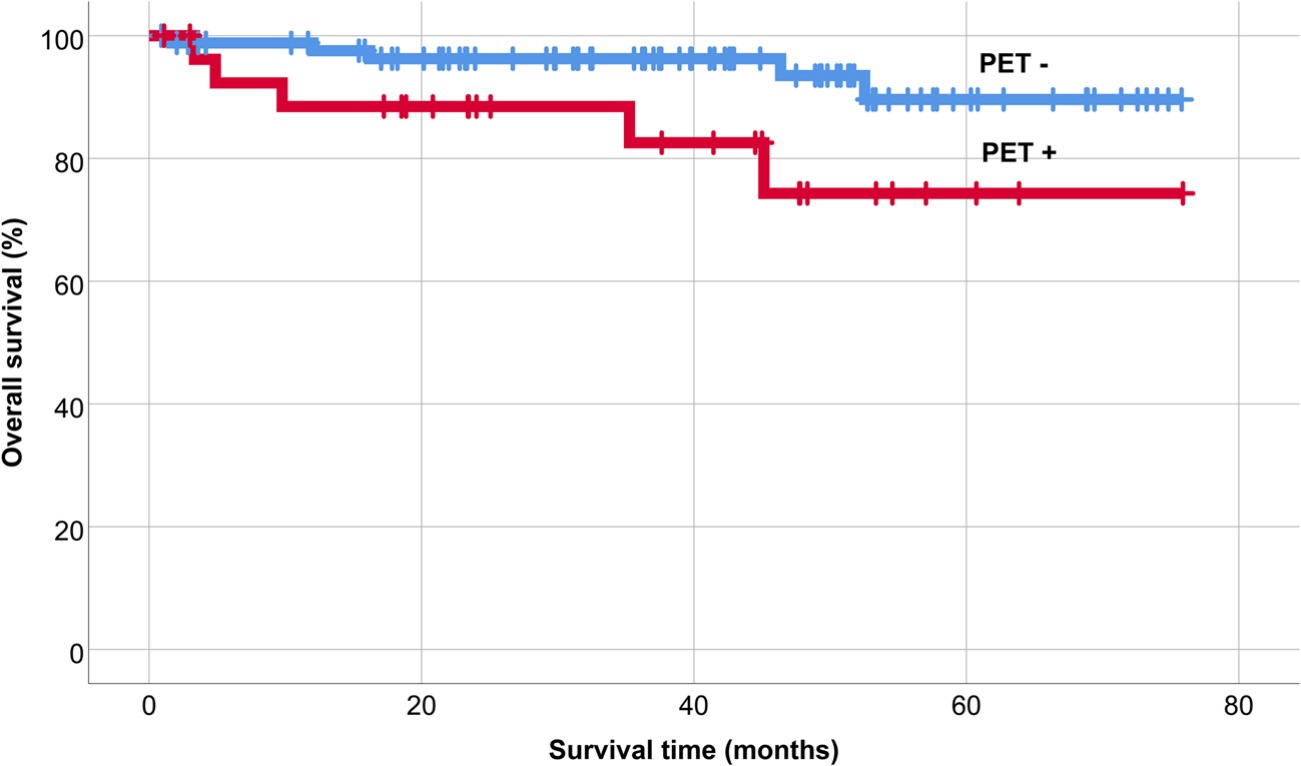
Overall survival based on the result of pretransplant PET/CT

**Fig. 3:**
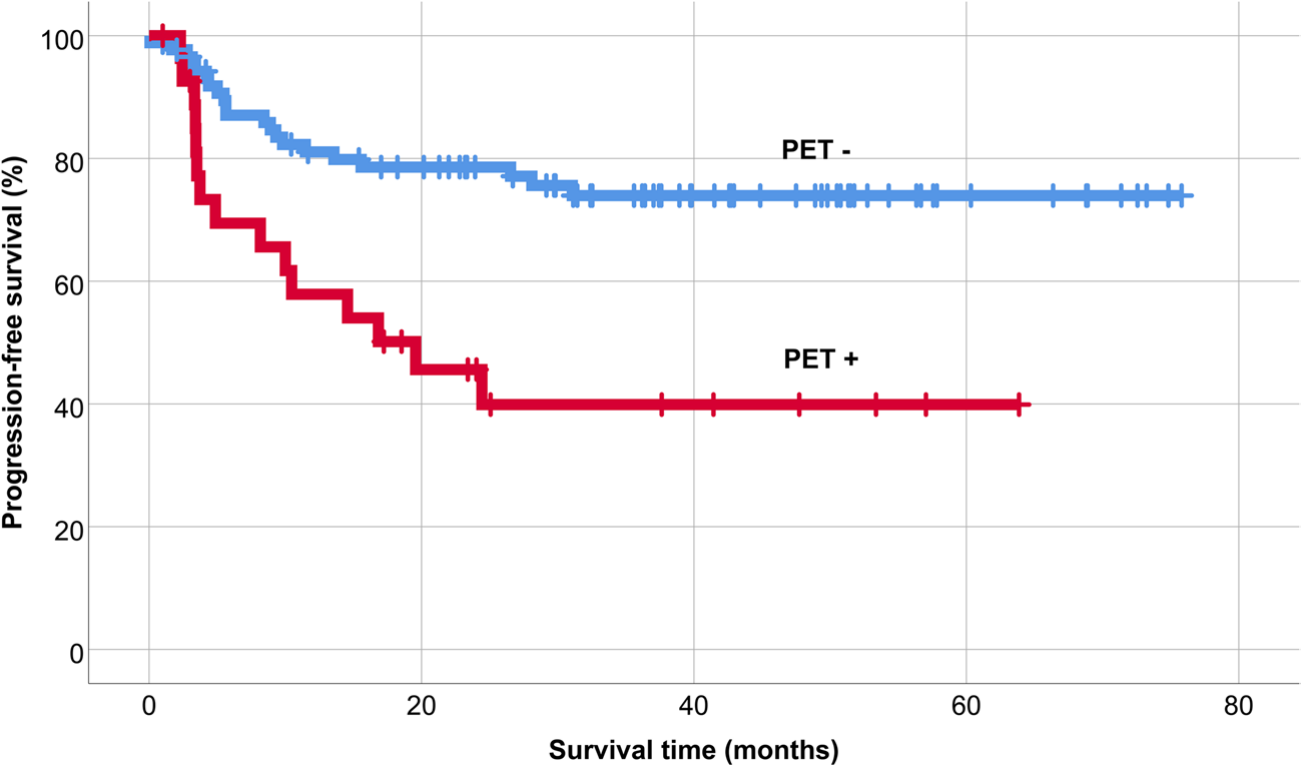
Progression-free survival based on the result of pretransplant PET/CT

**Fig. 4:**
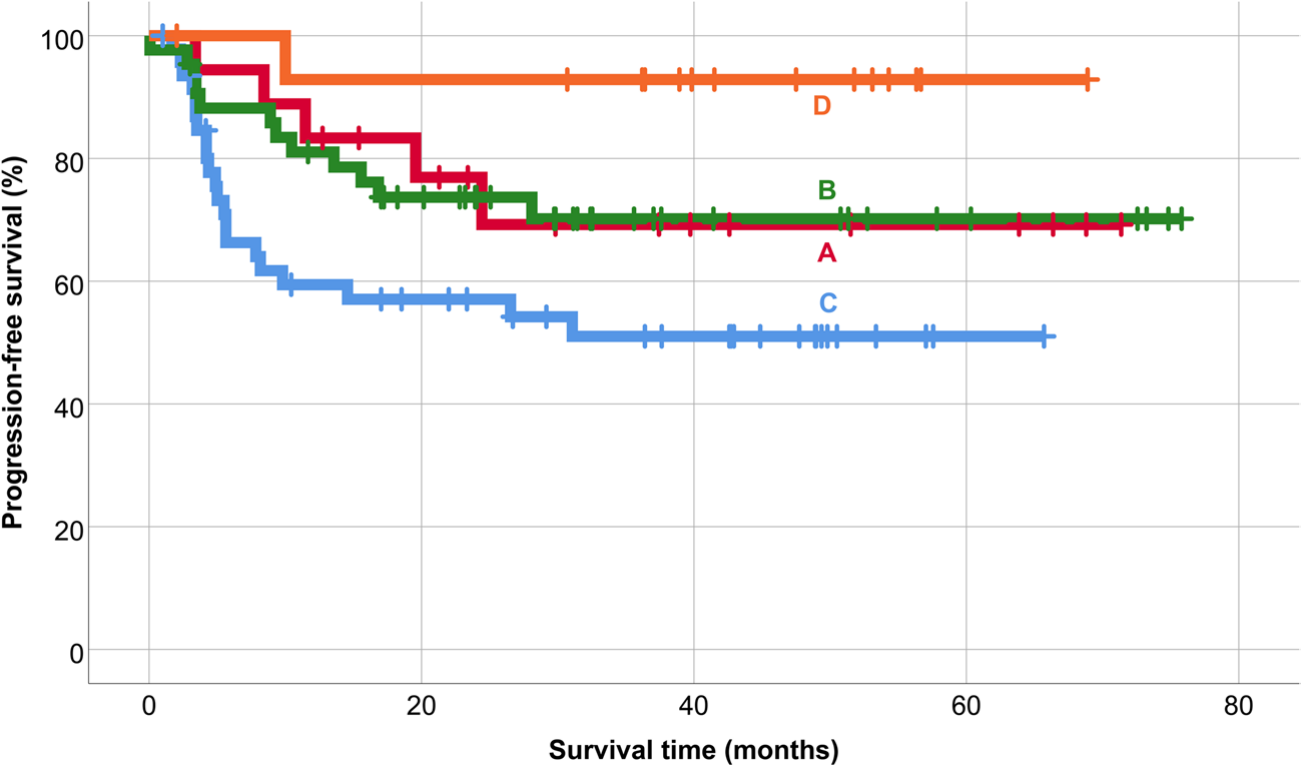
Progression-free survival based on the indication of brentuximab vedotin (BV) treatment (A, only BV maintenance therapy after AHSCT; B, both BV-based salvage therapy and BV maintenance therapy; C, only BV-based salvage therapy before AHSCT without BV maintenance; D, no BV treatment at all)

The 5-year overall survival after AHSCT was 86%, and 5-year PFS was 66% in the patient population (Fig. 1). PET/CT scan-positivity before AHSCT led to an inferior outcome (Figs. 2 and 3). The 5-year OS and PFS of PET/CT negative patients undergoing AHSCT were superior, compared to patients with metabolically active tumor burden after most recent salvage therapy (90% vs. 74%, *p* = 0.039, and 74% vs. 40%, *p* = 0.001, respectively). Relapse rates were also favorable in PET/CT scan negative patients, with 23% of them experiencing relapse post-AHSCT, compared to 43% among the PET/CT scan-positive population (*p* = 0.005). Altogether, pre-AHSCT BV therapy did not affect the outcome of HL patients. There was no difference in the 5-year OS or PFS between BV-recipients and those who did not receive BV-based salvage therapy before AHSCT (89% vs. 83%, *p* = 0.466, and 69% vs. 61%, *p* = 0.4, respectively). Regarding subgroups determined by not just the presence but the indication of BV treatment (only BV maintenance therapy after AHSCT, both BV-based salvage therapy and BV maintenance therapy, only BV-based salvage therapy before AHSCT without BV maintenance, and no BV treatment at all), there was no difference in the 5-year OS (89%, 93%, 75.5%, and 92%, respectively; *p* = 0.654). However, the 5-year PFS of these subgroups was different (69%, 70%, 51%, and 93%, respectively; *p* = 0.019) (Fig. 4).

The 18 patients (14%), who did not receive BV treatment at all, were at low risk based on the modified AETHERA criteria. Among them, no relapse occurred, and only one patient died due to alcoholic cardiomyopathy. In addition, high-risk patients who received BV-maintenance after AHSCT (whether they received BV before AHSCT or not) experienced superior PFS compared to those who did not continue with BV-maintenance treatment after AHSCT (*p* = 0.031). A total of 35 patients (27%) relapsed after AHSCT, 25 (71%) of them within 12 months, and 7 further (5%) within 24 months. Twenty-two patients out of 28 patients undergoing AHSCT with PET/CT-positivity underwent PET/CT during the first follow-up visit scheduled 100 days post-procedure. Half of them (55%) presented without metabolically active tumor burden. Twenty patients who relapsed after AHSCT received programmed cell death-1 (PD1) inhibitor treatment. Application of PD1 inhibitors is not routinely financed by the National Health Insurance Fund of Hungary (NHIFH). Pre-transplant (pembrolizumab) or post-transplant (nivolumab and pembrolizumab) application of checkpoint inhibitors depends on previous ineffectivity or intolerance of brentuximab vedotin and application of an individual aid request by the NFIFH. A total of 11 patients died; however, only 2 deaths were due to the progression of HL. We lost 5 further patients to HL-related, non-relapse mortality, while 2 patients died of unrelated causes to HL. 

## Discussion

Approximately half of the R/R HL patients relapse after AHSCT [3–9]. The success of AHSCT is influenced by many prognostic factors (primary chemorefractory disease, stage IV at relapse, extranodal involvement, B symptoms, depth of remission before transplantation). However, the hallmark of the management of candidates for AHSCT in HL is the achievement of CR after salvage therapy [3, 5, 6, 12, 16–18].

Moskowitz and colleagues examined the prognostic role of PET/CT before AHSCT in a phase II trial of 97 HL patients. The event-free survival (EFS) of PET/CT-negative patients was above 80%, compared to 29% in patients with PET/CT positivity [19]. Today, PET/CT is routinely used before AHSCT, and achieving a complete metabolic response is the primary goal of salvage treatment [10, 20, 21]. Our results confirm the importance of the total absence of active disease in this setting, proving that both OS and PFS are superior in patients with PET/CT negativity at the time of AHSCT.

CR rate is between 17 and 75% with traditional salvage treatments (DHAP, ESHAP, IGEV, ICE) [21], but the potential for achieving CR has expanded with the advent of targeted therapies (BV, PD1 inhibitors). Single-agent BV as a salvage therapy has a 34% CR rate and 75% overall response rate (ORR) in heavily pretreated patients [22], while different BV-based combined therapies (BV-Bendamustin, BV-DHAP, BV-ESHAP, BV-gemcitabine, BV-ICE) presented far superior remission rates (CR 69–81%, ORR 74–95%) [23]. The outcome of AHSCT, chances of recovery, and survival of those patients who relapse after transplantation are also improved by incorporating these treatment options into HL therapy.

The AETHERA trial proved that maintenance therapy with brentuximab vedotin improves PFS, compared to placebo, in patients at high risk for relapse after AHSCT. The 5-year PFS was 59% in the BV group, compared to 41% in the placebo group. Altogether, 33% of these patients relapsed, 20% of them within the first year. By the nature of the study, there were no BV-pretreated patients in the AETHERA trial. However, since then, BV has been incorporated into pre-ASCT salvage treatments and emerged to the first-line setting in advanced stage HL based on the results of the ECHELON-1 trial.

A few clinical studies have confirmed the improved survival after AHSCT, but there was a difference between studies based on the use of BV [23–29].

As a result of this process, the proportion of HL patients receiving BV therapy before AHSCT is growing by the day. Veilleux et al. analyzed the data of 89 R/R HL patients who underwent AHSCT between 2007 and 2019 [23]. The median follow-up was 5 years, and the estimated 5-year PFS and OS were 57.5% and 81.3%, respectively. Before AHSCT, 61% of patients had CR, 27% had PR, and 7% had progressive disease; pretransplantation PET was negative (Deauville score 1–3) in 61%. Only 9 patients were receiving BV treatment before AHSCT. The cumulative incidence of relapse was 34%, with 18 patients deceased [23]. Akay et al. investigated 75 patients who underwent AHSCT between 2016 and 2019 [24]. All patients received BV consolidation after AHSCT, and 23% received BV therapy before AHSCT. The median follow-up was 26 months, the 2-year OS was 87.6%, and 2-year PFS was 67.7%. Ten patients died, 8 from HL and two from infectious complications. Most (64.7%) patients were in CR at the time of AHSCT, while the PET/CT scan was negative (Deauville score 1–3) in only 43%. There was no difference in survival rates between those who received BV before transplantation and those who did not.

The study of Marouf et al. presented a similar result after analyzing the data of 115 patients with HL undergoing AHSCT [25]. The 2-year OS and PFS were 96.4% and 75.3%. Remission rates before AHSCT were higher compared to other studies (Table 2). Twenty percent of patients did not have centrally reviewed PET/CT before transplantation. The depth of remission was only known in patients who had centrally reviewed PET/CT, which probably explains the slightly better remission rate than our results. Seventy percent of the 115 patients received BV before AHSCT, and there was no difference in PFS regarding BV administration. These observations are consistent with our results. Also, in this report, 55.65% of patients received ABVD and 36.5% escBEACOPP, with 2-year PFS, OS, and relapse rates similar to our study.

**Table 2:** The present study compared with other studies

Study	Time period	Number of patients	PFS	OS	BV before AHSCT (number of patients)	Pre AHSCT PET negative status	Post AHSCT remission rate	BV maintenance treatment (number of patients)	Relapse
Roerden et al. 2020 [31]	1996–2017	66	46.1%	59.5%	2	93.7%*	Unknown	0	48.5%
			(5 year)	(5 year)					
Veilleux et al. 2021 [23]	2007–2019	72	57.5%	81.3%	9	88%	92%	0	34%
			(5 year)	(5 year)		61%			
Fakhri et al. 2021 [32]	2007–2017	218	52%	78%					
			(4 year)	(4 year)	22	51%	Unknown	43	Unknown
Massaro et al. 2021 [29]	2011–2020	105	62%	86%					
			(3 year)	(3 year)	54	75%	78%	105	29%
Akay et al. 2021 [24]	2016–2019	75	67.75% (2 year)	87.61% (2 year)	17	43%	93.3%	75	33.33%
Marouf et al. 2020 [25]	2012–2017	115	75.3%	96.4%	81	82.4%	Unknown	115	26%
			(2 year)	(2 year)					
Martinez et al. 2022 [30]	2013–2021	156	70%	91.6%	57	64.7%	Unknown	62	32.7%
			(3 year)	(3 year)					
Shah et al. 2021 [28]	2000–2009	99	49.5%	65% (4 year)	0	73%	Unknown	0	43%
	2010–2018	110		80% (4 year)	31	91%	Unknown	18	34%
Chung et al. 2021 [26]	2011–2015	51	56% (5 year)	86% (5 year)	10	87.2%*	91.2%*	4	Unknown
	2015–2020	63				86.9%*	79.1%*	45	
Spinner et al. 2023 [27]	2001–2010	159	63.3% (4 year)	79% (4 year)	0	42%	Unknown	0	30.5%
	2011–2020	183	73.1% (4 year)	89.1% (4 year)	78	61%		25	23.9%
Present study 2023	2016–2020	126	73%	94%	86	76%	77%	61	27%
			(2 year)	(2 year)					
			66%	86%					
			(5 year)	(5 year)					

*BV*, brentuximab vedotin; *PFS*, progression-free survival; *OS*, overall survival; *AHSCT*, autologous stem cell transplantation. *Complete and partial remission, PET negativity unknown

Chung et al. demonstrated similarly good overall survival as we did. Although the exact number of their patients receiving BV pre-AHSCT is not known in the 2015–2020 period, and post-AHSCT BV was used in 71% of patients, which is a higher proportion compared to our data [26].

Spinner et al. [27] and Shah et al. [28] similarly demonstrated a significantly improved OS comparing patients transplanted after 2010. Contrary to our data, the proportion of patients receiving BV (both before and after AHSCT) was lower.

The vast majority of our patients in this study received BV treatment before or after transplantation or both. The number of patients receiving maintenance therapy alone was relatively low. Nearly half of the patients treated with a BV-based salvage regimen before AHSCT have also received BV maintenance therapy. We did not observe any difference in the outcome of patients who proceeded to AHSCT with or without prior BV treatment.

There was difference in progression-free survival rates between high-risk patients who received BV maintenance treatment and those high-risk patients who did not get, but there was no survival difference, who did not require such treatment. Therefore, in our opinion, this result can be considered a validation of the indications of post-AHSCT BV therapy established via the AETHERA trial in a real-world setting.

Patients who received one line of salvage therapy before AHSCT had no survival benefit compared to patients treated with two or more salvage regimens. This observation is in line with the results of the study by Massaro and colleagues [29]. The incidence of post-AHSCT relapse was lower compared to prior studies, which can be accounted for the higher proportion of BV therapy before AHSCT. Based on historical data, 71% of relapses occur within 1 year after AHSCT, and 90% of HL recurrence is observed within 2 years [3, 26]. Our present study confirms this observation. In Martinez et al.’s study of 156 high-risk HL patients, 62 patients received BV consolidation treatment [30]. The 3-year OS and PFS were 91.6% and 70%. PFS was significantly better in the group with BV consolidation therapy than without (*p* = 0.004). The 3- year PFS was 52.5% (BV therapy only before AHSCT), 64.4% (no BV treatment at all), 80.5% (only BV consolidation treatment), and 88.9% (BV before and after AHSCT). 36.5% of patients received BV before transplantation, 34.6% were treated ≥ 2 or more salvage treatments, and 64.7% had negative PET/CT before AHSCT [27]. These results are similar to our results, but in our study we did not only analyze data of high-risk patients. Our goal was to achieve PET negativity before transplantation, so more patients received ≥ 2 or more salvage treatments among our patients and they were treated with BV before AHSCT (68%), and 76% of all patients reached PET negativity before transplantation. While a small proportion of the deaths were due to the progression of HL, the treatment-related mortality rate highlights the importance of supportive care in this setting.

In the majority of publications analyzing a similar group of patients, ABVD was the first-line treatment choice by a dominant margin [23–25, 27, 29–32]. Several studies have confirmed that ABVD is the most commonly used first-line treatment in many European countries and in North America [33–35]. This is explained by the favorable toxicity profile associated with excellent long-term outcome, as well as the lack of alternatives promising better OS. Undoubtedly, BEACOPP protocols are more effective regarding short-term PFS, an indicator that plays a more important role in the German perspective [34].

The complex task of balancing efficacy in disease control with the risk of side effects in Hodgkin lymphoma (HL) often leads to variations in clinical practice. The utilization of BEACOPP and escBEACOPP protocols is generally limited to well-defined, high-risk patients (Hasenclever-Diehl score > 4, advanced stage HL). However, in our region, these protocols have not been widely adopted due to concerns regarding both early and late side effects (e.g., early toxicity, incidence of secondary malignancies, and infertility issues), as well as challenges in procuring procarbazine. Notably, HL predominantly affects young and working-age individuals, making the incidence of secondary malignancies and infertility problems associated with BEACOPP protocols a critical concern for this specific social group. This challenge becomes more pronounced in light of the growing availability of modern biological therapies, which offer significantly lower toxicity profiles. Overall, the future role of BEACOPP in HL treatment is largely uncertain [36].

We acknowledge the limitations of this non-randomized study, including the retrospective nature of data collection and the relatively low number of patients receiving only BV maintenance therapy. However, in our opinion, real-world data is of particular importance in patient populations with unusual disease settings. Also, the inclusion of patients from all age groups with no regard to co-morbidities can be considered the main strength of the current report. To adequately attribute any outcome to the administration of BV in the pre-AHSCT setting, further information needs to be acquired in the form of sufficiently powered randomized control trials.

In our present study, the recovery rates of our R/R HL patient population, who underwent AHSCT, improved significantly. Our positive results can be attributed to the PET/CT directed, response-adapted treatment approach, and the widespread use of BV.

In conclusion, our results strongly underline the importance of achieving CR before AHSCT in HL, regardless of the number and type of previous treatments. The depth of remission before AHSCT affects the success of transplantation and the patients’ chances of recovery. PET negativity is an extremely important prognostic factor before transplantation, so this should always be the main goal of therapy, even with the use of novel agents (brentuximab vedotin, PD1 inhibitors). If the patient is not PET-negative before transplant, clinicians are urged to plan the post-transplant treatment (adjuvant radiation, brentuximab vedotin, PD1 inhibitors, or clinical trials) before or at the time of transplantation. Furthermore, AHSCT can provide prolonged disease control, which can be improved with the adequate use of BV therapy. It is important to use maintenance brentuximab vedotin treatment if the patient is at high risk of relapse, based on the modified AETHERA criteria. 
